# The characteristics of the mechanoreceptors of the hip with arthrosis

**DOI:** 10.1186/1749-799X-6-58

**Published:** 2011-11-16

**Authors:** Miguel RB Moraes, Maria LC Cavalcante, José AD Leite, José N Macedo, Marianna LB Sampaio, Vagnaldo F Jamacaru, Mariana G Santana

**Affiliations:** 1 Post-Graduate Departament of Surgery, Federal University of Ceará, Faculty of Medicine, 1608, Costa Mendes Professor St., 3rd floor, Rodolfo Teófilo, Fortaleza, 60530-140, Brazil

## Abstract

Mechanoreceptors have been extensively studied in different joints and distinct signals that convey proprioceptive information to the cortex. Several clinical reports have established a link between the number of mechanoreceptors and a deficient proprioceptive system; however, little or no literature suggest concentration of mechanoreceptors might be affected by hip arthrosis. The purpose of this study is first to determine the existence of mechanoreceptors and free nerve endings in the hip joint and to distinguish between their conditions: those with arthrosis and without arthrosis. Samples of 45 male hips were analyzed: 30 taken from patients with arthrosis that were submitted to total arthroplasty and 15 taken from male cadavers without arthrosis. The patients' ages ranged from 38 to75 years (average 56.5) and the cadavers' ages ranged from 21 to 50 years (average 35.5). The capsule, labrum, and femoral head ligament tissues were obtained during the arthroplasty procedure from 30 patients with arthrosis and from 15 male cadavers. The tissue was cut into fragments of around 3 mm. Each fragment was then immediately stained with gold chloride 1% solution and divided into sections of 6 μm thickness. The Mann-Whitney test was used for two groups and the ANOVA, Friedman and Kruskal-Wallis tests for more than two groups. Results show the mechanoreceptors (Pacini, Ruffini and Golgi corpuscles) and free nerve endings are present in the capsule, femoral head ligament, and labrum of the hip joint. When all the densities of the nerve endings were examined with regard to those with arthrosis and those without arthrosis, the mechanoreceptors of cadavers without arthrosis were found to be more pronounced and an increase in free nerve endings could be observed (p = 0.0082). Further studies, especially electrophysiological studies, need to be carried out to clarify the functions of the mechanoreceptors in the joints.

## Background

The proprioceptive system preserves the integrity and stabilizes the joints. It includes peripheral mechanoreceptors that detect distinct signals and convey the proprioceptive information to the cortex. These afferent and efferent feedback systems help to improve coordination of movement and posture thus prevent injuries from occurring. This function represents the first line of action taken by the mechanoreceptors and free nerve endings with regard to the ligament, muscle joints, and capsules [1,2].

In 1874, Rauber became the first scientist to identify the Pacini corpuscle in the human capsule [3]. Since then, mechanoreceptors have been extensively studied in different joints [4-14]. However, only a few investigators have carried out comparative studies of the concentration of mechanoreceptors in the hip [15-17]. A correlation of the number of nerve endings and the deficit of the proprioceptive system has been found in joint diseases. The performance of the proprioceptive system affects joint stability and can be a contributory cause of lesion of the cartilage [10,12,18,19].

This study has identified and quantified the mechanoreceptors and free nerve endings in the femoral head ligament, labrum, and capsule joint. These structures serve to stabilize hip joints. The density was measured and compared in 30 arthrosis and 15 normal hips joints. The morphological features were based on Freeman and Wyke's classification [20]. This research has a significant clinical application because proprioceptive training plays an important role in the prevention and treatment of orthopedic lesions.

## Methods

Forty-five hips were analyzed both from male patients with advanced arthrosis who had been submitted to total arthroplasty and from male cadavers. 30 hips were obtained from patients with arthrosis during the arthroplasty procedure. The ages ranged from 38 to 75 years (SD 56.5). Fifteen hips were from cadavers without arthrosis. The ages ranged from 21 to 50 years (SD 35.5).

Radiographs were taken before the tissue was removed and the degree of arthrosis examined on the basis of Bombelli's classification [21]. This study was approved by Ethics Committee No. 007.06.01 of the Federal University of Ceara.

**Figure 1:**
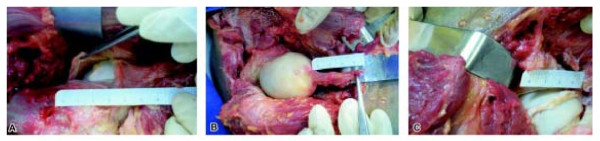
**Pictures showing the structures (A) Articular capsule hip (B) Femoral head ligament C) Acetabular labrum**.

An incision was made in the hips by means of the Watson Jones' approach as well as by employing the arthroplasty procedure, of which the capsule, labrum and femoral head ligament tissues were removed (Figure 1). Following this, the tissue was cut into fragments of around 3mm. Each fragment was immediately stained with 1% solution of gold chloride and divided into sections of 6 μm thickness. These sections were viewed through a light microscope [22].

**Figure 2:**
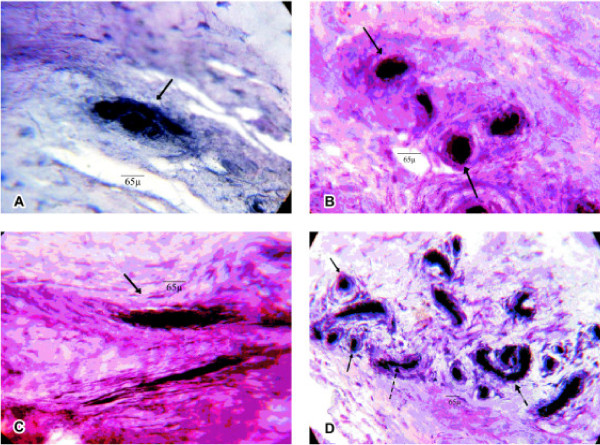
**Microscopy optical images with (A) Ruffini's corpuscle (400x) (B) Pacini's corpuscle (400x) (C) Golgi's corpuscle (400x) (D) Zimny method with goldchloride 1% solution**. Only in (D) arrows pointing Pacini corpuscle (→), Free nerve ending (----›) and Golgi corpuscle (— □ □›) (100x).

Four types of nerve endings were based on Freeman and Wike's classification: Type I (Ruffini) low-threshold and slow adapting; Type II (Pacini) low-threshold and fast adapting; Type III (Golgi) low-threshold and slow adapting; and Type VI (Free nerve ending) high-threshold, nocireceptors (Figure 2). A histomorphometry evaluation was undertaken and the density was determined by means of the point-counting method (40/400×) [23,24].

### Statistical Analysis

The Kolmogorov-Smirnov (ks) test was applied to all categories. The parametric data were measured by using mean and standard deviation. The non-parametric test included the quartile interval, and the minimum and maximum median values. The statistical method employed for making a comparison between the two groups was the Mann-Whitney Test. When there were more than two groups, the ANOVA, Friedman and Kruskal-Wallis tests were applied. When all the groups were compared, the difference between them was significant when p was less than 0.05 (Graphpad prism software 5.00; San Diego, CA; http://www.graphpad.com).

## Results

With regard to the 15 cadaveric hips without arthrosis, the histological evaluation of the capsule, femoral head ligament, and labrum acetabular showed that the tissue had distinctive characteristics. The joint capsule showed the presence of dense conjunctive tissue, a few conjunctive cells and fibroblasts. In addition, there were parallel and abundant collagen fibers.

The femoral head ligament showed the presence of superficial collagen fibers and was in a longitudinal direction. The deep collagen fibers showed signs of disorganization and an increased number of vessels. The acetabular labrum had thick and parallel collagen.

There was a reduction in the number of collagen fibers and vessels in the arthrosis group. However, there were no morphological differences between the mechanoreceptors in each group.

In both groups, arthrosis and normal hip, the Ruffini corpuscles appeared to be globular ramifications with a diameter of around 100 mμ. The Pacini corpuscles had a spherical shape with external lamellas and measured 50 - 100 mμ. The Golgi corpuscles proved to be bigger (up to 400 mμ) and had a helical shape, with long spindles. The free nerve endings were fine and without any set pattern.

**Figure 3:**
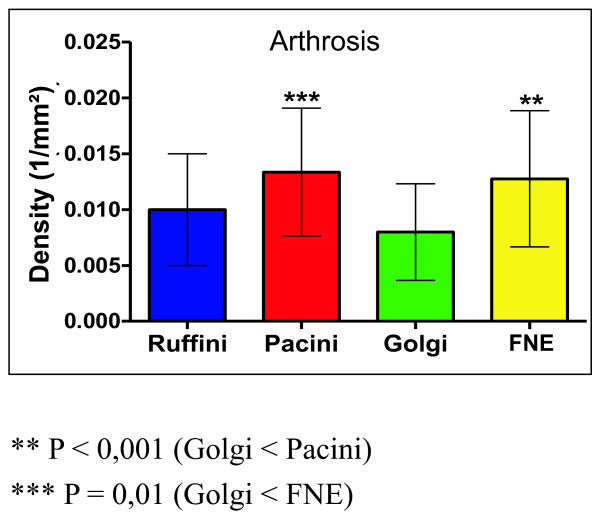
**Total density of the mechanoreceptors in hip with arthrosis**.

**Figure 4:**
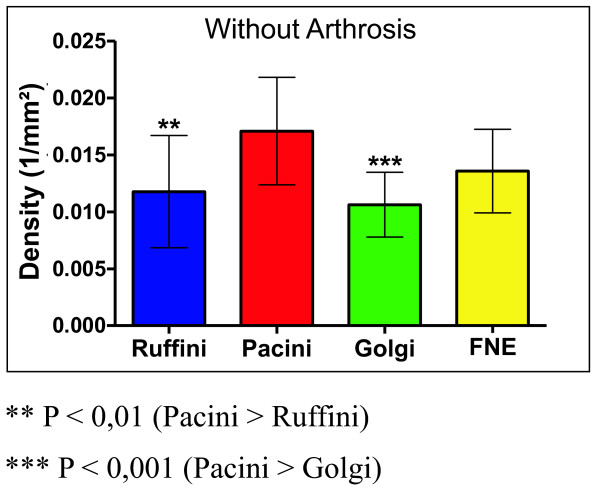
**Total density of the mechanoreceptors in hip without arthrosis**.

In the case of the patients with arthrosis, there was a significant reduction of Golgi corpuscles (0.008/mm2) when compared with Pacini corpuscles (0.013/mm2) (P < 0.001) and free nerve endings (0.012/mm2) (P < 0.01) (Figure 3 and table 1). However, in the group without arthrosis, there was a significant increase in the Pacini corpuscle's density (0.017/mm2) when compared with Ruffini (0.012/mm2) (P < 0.01) and Golgi (0.011/mm2) (P < 0.001) corpuscles (Figure 4 and table 2).

**Table 1:** Total density of the mechanoreceptors in hip with arthrosis

Mechanoreceptor	Arthrosis	
	**Mean**	**SD**
Ruffini	0,010	0,005
Pacini	0,013	0,006
Golgi	0.008	0,005
FNE	0,012	0,006

Note: FNE = nerve free ending, SD = standard deviation

**Table 2:** Total density of the mechanoreceptors in hip without arthrosis

Mechanoreceptor	Without arthrosis	
	**Mean**	**SD**
Ruffini	0,012	0,005
Pacini	0,017	0,005
Golgi	0,011	0,002
FNE	0,013	0,004

Note: FNE = free nerve ending, SD = standard deviation

**Figure 5:**
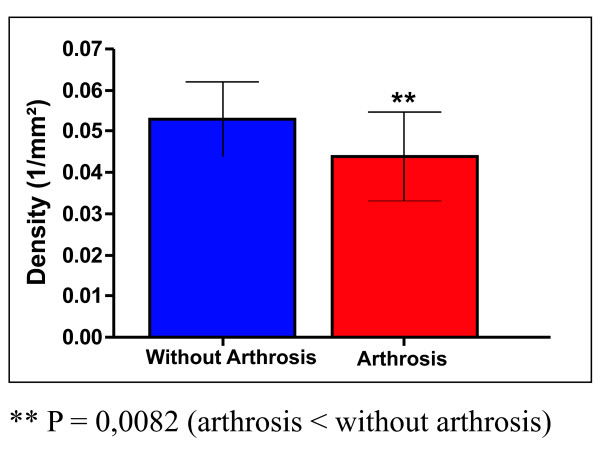
**Total density of the mechanoreceptors in hip without arthrosis and with arthrosis**.

When the total number of nerve ending densities were compared between patients with arthrosis and those without arthrosis, the mechanoreceptors of the cadavers without arthrosis were found to be more pronounced and a decrease in the number of the nerve endings could be observed among the patients with arthrosis (P = 0.0082) (Figure 5 and table 3).

**Table 3:** Total density of the mechanoreceptors in hip without arthrosis and with arthosis

Without arthrosis		Arthrosis	
Mean	SD	Mean	SD
0,053	0,007	0,044	0,011

Note: SD = standard deviation

## Discussion

Mechanoreceptors have been identified in structures such as capsule, ligament, and fibrocartilage tissues from human and animal specimens [2,4,6,11,25-27]. There has been an increase in the status of mechanoreceptors in orthopedic diseases and this has led to a great deal of research into the alterations that occur in the joints [4,9,10,12,28]. However, no references have been found in the literature of comparative studies between patients with or without arthrosis in the hip.

Currently, investigators are conducting morphological and electrophysiological studies of these structures. In the current study, a histomorphological analysis was described that allowed us to visualize mechanoreceptors and free nerve endings and distinguish them in different conditions between subjects with and without arthrosis.

Gold chloride was used to stain the mechanoreceptors to allow each structure to be distinguished. This technique was employed by Amir, Cavalcante and Michelson [4,5,13] to identify cells, collagens, fascicular regions and conjunctive tissue. The immunohistochemical has revealed further details, although at a high cost [6,7,26,29-31].

The morphological features of the mechanoreceptors observed were similar to those identified by Freeman and Wyke [20] and it also was related by others authors when they used the same classification to describe elbow ligaments [11], sinus tarsi syndrome [32] and ruptured knee ligaments [33].

Mechanoreceptors were found in three structures that serve to stabilize the hip joint: the capsule, femoral head ligament, and labrum and our experiments closely followed the work of most other investigators who have described nerve endings in the hip joint [15,16].

When the groups with and without arthrosis were compared, there was a significantly greater reduction in the Pacini type (P < 0.0351) than the Ruffini type (P = 0.2674). The Pacini corpuscles are low threshold and able to adapt quickly while the Ruffini corpuscles only adapt slowly [3,20]. Additionally, it means that there was a greater loss of nerve endings among those that adapted rapidly to the groups with arthrosis.

With regard to the total number of densities of the mechanoreceptors in the two groups, there was a significant reduction in the arthrosis group (P = 0.0082). Morisawa, Franchi, Muratli and Kontinen [10,12,28,34] also observed a decrease in the other disease joints. This is strong evidence that these structures play a significant role in the proprioceptive system. However, the amount of mechanoreceptors present could be affected by factors such as hip diseases, in addition to the proprioceptive system and stability of the joints. Our results showed that there was a considerable reduction of mechanoreceptors when the hip joint was subject to arthrosis.

Further studies, especially in electrophysiological areas, need to be carried out to clarify the functions of the mechanoreceptors in the joints, as the treatment of most orthopedic diseases is beginning to include programs for proprioceptive rehabilitation [27,35-41]. In the future, people who have slow reflexes, lax joints, joint incongruity, and loss of muscle power will benefit from improvements in proprioception.

## Conclusion

The study of mechanoreceptors is important because it improves knowledge about the proprioception system and helps to develop an efficient rehabilitation program.

## Competing interests

The authors declare that they have no competing interests.

## Authors' contributions

MRBM, conceived and carried out the experiment; MLCC, JADL, participated in the analysis of the study and its supervision; JNM, helped to select the patients, and participated in the surgery; VFJ, conducted the statistical analysis; MLBS, MGS, participated in the laboratory analysis. All the authors read and approved of the final manuscript
